# Circulating but not faecal short-chain fatty acids are related to insulin sensitivity, lipolysis and GLP-1 concentrations in humans

**DOI:** 10.1038/s41598-019-48775-0

**Published:** 2019-08-29

**Authors:** Mattea Müller, Manuel A. González Hernández, Gijs H. Goossens, Dorien Reijnders, Jens J. Holst, Johan W. E. Jocken, Hans van Eijk, Emanuel E. Canfora, Ellen E. Blaak

**Affiliations:** 1 Department of Human Biology, NUTRIM School of Nutrition and Translational Research in Metabolism, Maastricht University Medical Centre+, Maastricht, The Netherlands; 2 Department of Pediatrics, Maastricht University Medical Centre+, Maastricht, The Netherlands; 3 NNF Center for Basic Metabolic Research and Department of Biomedical Sciences, Faculty of Health and Medical Sciences, University of Copenhagen, Copenhagen, Denmark; 4 Department of Surgery, NUTRIM School of Nutrition and Translational Research in Metabolism, Maastricht University Medical Center+, Maastricht, The Netherlands

**Keywords:** Metabolic syndrome, Gastrointestinal hormones

## Abstract

Microbial-derived short-chain fatty acids (SCFA) acetate, propionate and butyrate may provide a link between gut microbiota and whole-body insulin sensitivity (IS). In this cross-sectional study (160 participants, 64% male, BMI: 19.2–41.0 kg/m^2^, normal or impaired glucose metabolism), associations between SCFA (faecal and fasting circulating) and circulating metabolites, substrate oxidation and IS were investigated. In a subgroup (n = 93), IS was determined using a hyperinsulinemic-euglycemic clamp. Data were analyzed using multiple linear regression analysis adjusted for sex, age and BMI. Fasting circulating acetate, propionate and butyrate concentrations were positively associated with fasting GLP-1 concentrations. Additionally, circulating SCFA were negatively related to whole-body lipolysis (glycerol), triacylglycerols and free fatty acids levels (standardized (std) β adjusted (adj) −0.190, P = 0.023; std β adj −0.202, P = 0.010; std β adj −0.306, P = 0.001, respectively). Circulating acetate and propionate were, respectively, negatively and positively correlated with IS (M-value: std β adj −0.294, P < 0.001; std β adj 0.161, P = 0.033, respectively). We show that circulating rather than faecal SCFA were associated with GLP-1 concentrations, whole-body lipolysis and peripheral IS in humans. Therefore, circulating SCFA are more directly linked to metabolic health, which indicates the need to measure circulating SCFA in human prebiotic/probiotic intervention studies as a biomarker/mediator of effects on host metabolism.

## Introduction

In obesity and type 2 diabetes mellitus (T2DM), alterations in the gut microbiota composition and functionality may contribute to disease aetiology. The gut microbiota ferments indigestible carbohydrates (e.g. dietary fibres) and major end-products thereof are the short- chain fatty acids (SCFA) acetate, propionate and butyrate^1^. Acetate, propionate and butyrate are present in the colon in a ratio of approximately 3:1:1, respectively^2,3^. Most butyrate is utilized by colonocytes as energy source^4^. Via the portal vein, SCFA reach the liver where acetate and propionate are metabolized and partly oxidised or used as substrate in gluconeogenesis and lipogenesis^5^. Consequently, a small proportion of microbial-derived SCFA enters the peripheral circulation whereby acetate reaches the highest concentrations compared to propionate and butyrate^6,7^. SCFA are ligands to G-protein coupled receptors (GPR) 41 and 43, which are expressed on intestinal, adipose, skeletal muscle, liver and pancreatic tissues^8–10^, indicating their important role in the crosstalk between the gut and peripheral tissues. Several rodent studies showed that oral, intravenous and colonic infusion of SCFA as well as microbial-derived SCFA beneficially affect the functioning and metabolism of the aforementioned tissues and consequently improve insulin sensitivity, substrate metabolism and body weight regulation^11^. In humans, distal colonic acetate infusions increased fasting fat oxidation, energy expenditure, and peptide YY (PYY) secretion whilst whole-body lipolysis was decreased^12,13^. Other acute studies show decreased circulating free fatty acid (FFA) concentration after rectal SCFA infusion in healthy participants^14,15^. In addition, 24 weeks of 10 g/day inulin propionate ester protected against body weight gain as compared to inulin only in overweight individuals^16^. Potential mechanisms may include a SCFA-induced inhibition of energy intake possibly mediated via the stimulation of glucagon-like peptide 1 (GLP-1) and PYY secretion, increased intestinal gluconeogenesis, increased skeletal muscle fat oxidation and improved lipid buffering capacity of adipose tissue^11^. However, increased microbial-derived acetate formation has been associated with increased body weight gain and insulin resistance in diet-induced obese rats^17^. Additionally, increased faecal SCFA have been reported in overweight and obese compared to lean participants^2,3,8,18,19^, yet it is difficult to interpret the latter data since faecal SCFA reflect the net result of colonic production and absorption^20,21^. Even though faecal SCFA are commonly used as an indicator of microbial fermentation, faecal SCFA may not accurately reflect *in vivo* colonic fermentation since approximately 95% of colonic SCFA are absorbed and only the remaining 5% are excreted in feces^22–25^.

To obtain more information on the validity of faecal SCFA as biomarker for metabolic health effects, the associations between faecal and circulating SCFA concentrations and parameters of metabolic health were studied in a relatively large cohort of 160 participants with a wide range of body mass indices (BMI) and glucometabolic status. Using multiple regression analysis, we analysed the relationship between faecal and fasting circulating SCFA with fasting glucose, insulin, circulating lipids (free fatty acids (FFA), triacylglycerols (TAG), glycerol), insulin resistance index (homeostasis model assessment of insulin resistance (HOMA-IR)), gut hormone concentrations (PYY, GLP-1), fasting substrate utilization and inflammation markers including lipopolysaccharide-binding protein (LBP), tumour necrosis factor alpha (TNF-α), interleukin 6 (IL-6) and interleukin 8 (IL-8). We further investigated the relationship between faecal and fasting circulating SCFA profiles and peripheral insulin sensitivity index (M-value) as measured via the gold standard hyperinsulinaemic-euglycemic clamp technique.

## Results

Mean age of the participants was 49.6 ± 14.7 years and 66.2% of participants were male with a mean BMI of 29.8 ± 4.4 kg/m^2^, a mean fasting glucose of 5.6 ± 0.6 mmol/L and a mean HOMA-IR of 3.7 ± 1.5 (Table 1). In the subgroup, peripheral (M-value) was measured in 93 overweight to obese, prediabetic men (n = 72) and women (n = 21) with mean age of 59.0 ± 7.1 years and a mean BMI of 31.8 ± 3.1 kg/m^2^, respectively.

**Table 1:** Characteristics of participants.

Characteristic	Mean ± SD	Range
Male (n)/Female (n)	106/54	
Age (y)	49.6 ± 14.7	20–69
Body Weight (kg)	90.8 ± 15.7	52.8–125.9
BMI (kg/m^2^)	29.8 ± 4.4	19.2–41.0
Waist:Hip ratio	0.93 ± 0.13	0.64–1.22
HOMA-IR	3.7 ± 1.5	1.0–9.0
Fasting plasma insulin (mU/L)	14.9 ± 9.4	2.4–82.7
Fasting plasma glucose (mmol/L)	5.6 ± 0.6	3.1–7.5
Fasting plasma free fatty acids (μmol/L)	672 ± 243	140.3–1620
Fasting plasma triacylglycerol (μmol/L)	1178 ± 613	311.0 – 3944
Fasting free glycerol (μmol/L)	102.1 ± 46	27.1–372.1
Plasma acetate (μmol/L)	65.2 ± 64.7	2.8–429.4
Plasma propionate (μmol/L)	2.0 ± 1.6	0.06–12.0
Plasma butyrate (μmol/L)	1.7 ± 1.8	0.07–6.7
Faecal acetate (μmol/g)	44.2 ± 16.1	6.80–102.8
Faecal propionate (μmol/g)	13.0 ± 7.9	2.53–71.2
Faecal butyrate (μmol/g)	10.9 ± 5.9	0.00–33.8
*Subgroup hyperinsulinaemic-euglycaemic clamp*		
Male (n) /Female (n)	72/21	
BMI (kg/m^2^)	31.8 ± 3.1	26.1–41
Age (y)	59.0 ± 7.1	72–69
Fasting glucose (mmol/L)	6.0 ± 0.5	5.0–7.5
2 h glucose (mmol/L)	7.2 ± 1.8	3.3–11.2
M-value (mg*kg^−1^*min^−1^)	3.6 ± 1.5	1.7–8.3

BMI body mass index, HOMA-IR Homeostasis model assessment of insulin resistance, M-value mean glucose infusion rate at steady state during insulinemic- euglycemic clamps.

### Associations between faecal and circulating SCFA concentrations

Faecal acetate and butyrate were not associated to their respective circulating concentrations, while faecal propionate was positively associated with circulating propionate (standardized (std) std β = 0.262, P = 0.002).

### Circulating, but not faecal, SCFA are associated with BMI

Faecal acetate was positively associated with BMI (std β = 0.245, P = 0.004), however, after adjustment for age and sex, the association was not significant anymore (std β adj = 0.092, P = 0.214). Faecal propionate and butyrate were not significantly associated with BMI with or without adjustment for age and sex (faecal butyrate std β = 0.162, P = 0.055, faecal propionate std β = 0.023, P = 0.746). In contrast, circulating butyrate and propionate were significantly inversely related to BMI, also after adjustment for age and sex (circulating butyrate std β = −0.599, P < 0.001, circulating propionate std β = −0.290, P < 0.001, Supplementary Figure 1 K,L). Circulating acetate was negatively associated with BMI (std β = −0.285, P < 0.001), which was not significant anymore after adjustment for age and sex (std β = −0.115, P = 0.108).

### Faecal SCFA were not related to metabolic parameters

None of faecal SCFA were significantly associated with fasting GLP-1, PYY, FFA, TAG, glycerol, glucose, insulin concentrations, HOMA-IR, inflammatory markers or fasting substrate oxidation either with or without adjustment for age, sex and BMI. In the subgroup analysis faecal SCFA were not associated with peripheral insulin sensitivity (Table 2).

**Table 2:** Simple and multiple linear regression coefficients between faecal SCFA and metabolic parameters in fasting state.

*Metabolites*	Faecal acetate				Faecal propionate				Faecal butyrate			
	*std ß*	*P*	*std β adj*	*P*	*std ß*	*P*	*std β adj*	*P*	*std ß*	*P*	*std β adj*	*P*
FFA, μmol/L	0.052 ± 0.090	0.569	−0.048 ± 0.082	0.558	−0.098 ± 0.089	0.276	−0.092 ± 0.080	0.249	0.001 ± 0.090	0.991	−0.030 ± 0.081	0.707
TAG, μmol/L	0.058 ± 0.090	0.519	−0.057 ± 0.084	0.498	0.129 ± 0.089	0.151	0.037 ± 0.081	0.647	0.094 ± 0.090	0.296	−0.007 ± 0.082	0.931
Glycerol, μmol/L	−0.047 ± 0.090	0.603	−0.152 ± 0.086	0.079	−0.019 ± 0.090	0.836	−0.022 ± 0.084	0.797	−0.185 ± 0.145	0.205	−0.160 ± 0.084	0.059
***Hormones***												
Insulin, mU/L	0.275 ± 0.082	0.001	0.122 ± 0.071	0.088	0.185 ± 0.084	0.029	0.114 ± 0.069	0.101	0.207 ± 0.084	0.015	0.102 ± 0.070	0.146
GLP-1, pmol/L	−0.081 ± 0.092	0.379	0.114 ± 0.074	0.125	0.017 ± 0.092	0.852	0.140 ± 0.071	0.053	−0.037 ± 0.092	0.692	0.116 ± 0.072	0.113
PYY, pmol/L	−0.194 ± 0.106	0.070	−0.166 ± 0.112	0.140	−0.093 ± 0.107	0.386	−0.087 ± 0.110	0.428	−0.156 ± 0.106	0.146	−0.142 ± 0.110	0.199
***Insulin sensitivity***												
M-value, mg/kg/min	−0.071 ± 0.106	0.502	−0.035 ± 0.075	0.640	−0.029 ± 0.105	0.783	−0.050 ± 0.073	0.490	−0.100 ± 0.105	0.345	−0.111 ± 0.073	0.130
HOMA-IR	0.267 ± 0.084	0.002	0.080 ± 0.068	0.244	0.193 ± 0.085	0.025	0.091 ± 0.066	0.169	0.214 ± 0.085	0.013	0.076 ± 0.067	0.254
Glucose, mmol/L	0.174 ± 0.084	0.040	−0.029 ± 0.067	0.661	0.112 ± 0.084	0.187	0.006 ± 0.065	0.925	0.086 ± 0.085	0.310	−0.066 ± 0.066	0.315
***Inflammatory markers***												
LBP, pg/ml	0.152 ± 0.091	0.095	0.033 ± 0.086	0.699	0.024 ± 0.091	0.789	−0.010 ± 0.084	0.909	0.155 ± 0.090	0.088	0.092 ± 0.084	0.274
IL-6, pg/ml	0.164 ± 0.085	0.055	−0.010 ± 0.071	0.892	0.012 ± 0.085	0.887	−0.088 ± 0.069	0.204	0.039 ± 0.085	0.651	−0.094 ± 0.069	0.178
IL-8, pg/ml	0.157 ± 0.085	0.067	0.106 ± 0.088	0.231	0.164 ± 0.084	0.053	0.118 ± 0.085	0.170	0.115 ± 0.085	0.179	0.061 ± 0.086	0.479
TNF-α, pg/ml	0.196 ± 0.084	0.021	0.070 ± 0.078	0.170	0.063 ± 0.085	0.459	−0.046 ± 0.076	0.548	0.156 ± 0.084	0.067	0.039 ± 0.076	0.612
Substrate Oxidation												
Fat, E%	0.036 ± 0.092	0.691	0.001 ± 0.095	0.996	−0.002 ± 0.091	0.982	0.004 ± 0.093	0.970	0.013 ± 0.092	0.885	0.004 ± 0.094	0.969
CHO, E %	−0.092 ± 0.094	0.329	−0.084 ± 0.098	0.397	−0.034 ± 0.094	0.716	−0.042 ± 0.096	0.666	−0.110 ± 0.094	0.243	−0.116 ± 0.096	0.233

β, standardized β coefficient + standard error of coefficient of faecal acetate, propionate and butyrate as dependent variable in a simple regression and multiple regression analysis adjusted for age, sex and BMI. M-value (n = 93), PYY (n = 107). Adj, adjusted, std standardized, E%, percentage of energy expenditure, FFA, free fatty acids, TAG, triglycerides, GLP-1, glucagon-like peptide 1, PYY, peptide YY, HOMA-IR, homeostatic model assessment of insulin resistance, LBP, lipopolysaccharide binding protein, IL-6, interleukin 6, IL-8, interleukin 8, TNFα, tumour necrosis factor alpha.

### Fasting, circulating SCFA were related to fasting GLP-1, lipid metabolites and insulin sensitivity

All three circulating SCFA were positively associated with fasting GLP-1 concentrations (Table 3). Additionally, circulating acetate, propionate and butyrate were negatively associated with fasting glycerol, TAG and FFA, respectively. Also, circulating butyrate was negatively associated with fasting glucose. These relationships remained significant after adjustment for age, sex and BMI (Table 3, Supplementary Figure 1).

**Table 3:** Simple and multiple linear regression coefficients between circulating SCFA and metabolic parameters in fasting state.

*Metabolites*	Circulating acetate				Circulating propionate				Circulating butyrate			
	*std ß*	*P*	*std β adj*		*std ß*	*P*	*std β adj*		*std ß*	*P*	*std β adj*	*P*
FFA, μmol/L	−0.032 ± 0.084	0.701	0.003 ± 0.080	0.974	−0.302 ± 0.079	0.000	−0.127 ± 0.078	0.103	−0.381 ± 0.077	<0.001	−0.306 ± 0.090	**0.001**
TAG, μmol/L	−0.249 ± 0.081	0.003	−0.077 ± 0.081	0.340	−0.225 ± 0.081	0.006	−0.202 ± 0.078	**0.010**	−0.356 ± 0.078	<0.001	−0.137 ± 0.094	0.150
Glycerol, μmol/L	−0.204 ± 0.082	0.014	−0.190 ± 0.083	0.023*	−0.025 ± 0.083	0.768	0.155 ± 0.081	0.059	−0.286 ± 0.080	<0.001	−0.173 ± 0.098	0.079
***Hormones***												
Insulin, mU/L	−0.187 ± 0.079	0.019	0.003 ± 0.070	0.968	−0.234 ± 0.078	0.003	−0.066 ± 0.068	0.336	−0.406 ± 0.073	<0.001	−0.040 ± 0.082	0.625
GLP-1, pmol/L	0.402 ± 0.078	0.000	0.187 ± 0.070	**0.009**	0.318 ± 0.080	0.000	0.218 ± 0.068	0.002*	0.574 ± 0.069	<0.001	0.274 ± 0.081	**0.001**
PYY, pmol/L	0.125 ± 0.097	0.201	0.107 ± 0.107	0.317	0.080 ± 0.097	0.413	0.018 ± 0.105	0.868	0.182 ± 0.096	0.060	0.142 ± 0.126	0.263
***Insulin sensitivity***												
M-value, mg/kg/min	−0.224 ± 0.102	0.031	−0.294 ± 0.071	<**0.001**	0.327 ± 0.099	0.001	0.161 ± 0.074	0.033*	0.164 ± 0.103	0.113	−0.066 ± 0.091	0.469
HOMA-IR	−0.247 ± 0.079	0.002	−0.009 ± 0.067	0.892	−0.160 ± 0.080	0.047	0.007 ± 0.065	0.914	−0.461 ± 0.072	<0.001	−0.054 ± 0.078	0.494
Glucose, mmol/L	−0.228 ± 0.078	0.004	0.005 ± 0.066	0.939	−0.177 ± 0.078	0.025	−0.044 ± 0.064	0.499	−0.506 ± 0.068	<0.001	−0.200 ± 0.075	<**0.001**
***Inflammatory markers***												
LBP, pg/ml	−0.075 ± 0.088	0.399	0.038 ± 0.087	0.667	−0.262 ± 0.085	0.003	−0.117 ± 0.085	0.168	−0.330 ± 0.083	<0.001	−0.116 ± 0.102	0.254
IL-6, pg/ml	−0.298 ± 0.077	0.000	−0.098 ± 0.070	0.163	−0.177 ± 0.079	0.027	−0.027 ± 0.053	0.690	−0.406 ± 0.073	0.000	−0.021 ± 0.082	0.803
IL-8, pg/ml	−0.079 ± 0.080	0.324	0.024 ± 0.086	0.778	0.126 ± 0.079	0.113	0.164 ± 0.084	0.051	−0.023 ± 0.080	0.775	0.185 ± 0.100	0.067
TNF-α, pg/ml	−0.296 ± 0.076	0.000	−0.105 ± 0.076	0.168	−0.095 ± 0.079	0.232	−0.048 ± 0.075	0.523	−0.330 ± 0.075	<0.001	−0.047 ± 0.089	0.598
Substrate Oxidation												
Fat, E%	−0.156 ± 0.084	0.065	−0.021 ± 0.086	0.812	−0.117 ± 0.084	0.167	−0.052 ± 0.085	0.543	−0.317 ± 0.080	<0.001	−0.138 ± 0.101	0.172
CHO, E %	0.074 ± 0.087	0.396	0.159 ± 0.094	0.094	−0.092 ± 0.087	0.291	−0.167 ± 0.092	0.072	0.002 ± 0.087	0.986	0.047 ± 0.111	0.672

β, standardized β coefficient + standard error of coefficient of fasting circulating acetate, propionate and butyrate as dependent variable in a simple regression and multiple regression analysis adjusted for age, sex and BMI. M-value (n = 93), PYY (n = 107). Adj, adjusted, std standardized, E%, percentage of energy expenditure, FFA, free fatty acids, TAG, triglycerides, GLP-1, glucagon-like peptide 1, PYY, peptide YY, HOMA-IR, homeostatic model assessment of insulin resistance, LBP, lipopolysaccharide binding protein, IL-6, interleukin 6, IL-8, interleukin 8, TNFα, tumour necrosis factor alpha.

Circulating SCFA were not associated with fasting PYY, LBP, IL-6, IL-8 and TNF-α. Furthermore, circulating SCFA were not related to fat and carbohydrate oxidation, expressed as percentage of energy expenditure. In the subgroup analysis of overweight/obese, prediabetic individuals, peripheral insulin sensitivity was measured using the M-value derived from the euglycemic-hyperinsulinaemic clamp technique. We found that circulating acetate was negatively associated with peripheral insulin sensitivity (M-value) whereas circulating propionate was positively related to peripheral insulin sensitivity (Table 3, Supplementary Figure 1). The relationships between circulating SCFA and insulin sensitivity remained significant after adjustment for age, sex and BMI.

## Discussion

We investigated the relationship between faecal and fasting circulating SCFA with fasting plasma metabolites, gut hormones, substrate metabolism and inflammatory markers in a cohort with a wide range of BMI and glucometabolic status. This study shows that only circulating but not faecal SCFA concentrations were related to fasting plasma glucose, FFA, TAG and glycerol, GLP-1 and insulin sensitivity, also after adjustment for age, sex and BMI. Contrary to previous human studies, faecal SCFA were not related to BMI, whereas circulating butyrate and propionate were inversely associated with BMI. Circulating plasma propionate seems to be the most reflective of its respective faecal concentrations, whilst faecal acetate and butyrate were not related to their respective circulating concentrations. In line, previous literature reports that SCFA flux into the circulation and uptake in peripheral tissues rather than microbial SCFA production *per se* is of importance for metabolic health^26–28^. Our data emphasize the need to measure circulating SCFA in human prebiotic/probiotic intervention studies as a biomarker/mediator of effects on host metabolism.

To our knowledge, this is the first study providing evidence that fasting circulating SCFA are positively associated with fasting plasma GLP-1 in humans. High colonic SCFA production is linked to increased GLP-1 and PYY secretion through binding of SCFA to GPR41/43 on the enteroendocrine L-cell^29^. Further, a one-year dietary fiber intervention (wheat bran, 24 g/d) increased circulating SCFA concentrations accompanied by increased levels of GLP-1 concentrations in hyperinsulinemic participants^30^. Yet, there is little known about the contribution of circulating SCFA to GLP-1 secretion during the fasted state. Circulating SCFA may stimulate GLP-1 secretion from the visceral, basolateral side of enteroendocrine L-cells as observed in isolated rat colons^31^. Besides enteroendocrine L-cells, pancreatic α-cells have been suggested to contribute to systemic GLP-1 concentrations in the fasted state^32,33^, but whether circulating SCFA act as stimuli for GLP-1 secretion warrants further investigation.

In contrast to GLP-1, we did not find an association between circulating and faecal SCFA with fasting PYY. This is in contrast to human and *in vitro* studies reporting a stimulatory effect of SCFA on PYY secretion^12,34,35^, however to what extent SCFA and/or dietary fibres contribute to fasting PYY secretion remains to be investigated. Although the mechanisms still remain to be elucidated, the present data indicate that, despite being the net result of production, uptake and tissue utilization, circulating SCFA are more directly linked to metabolic health as compared to faecal SCFA.

In our study population, only circulating, but not faecal SCFA were associated with fasting plasma metabolites. Circulating acetate was negatively associated with fasting free glycerol, an indicator of whole-body lipolysis. This is consistent with *in vitro* and human *in vivo* studies reporting that acetate has an anti-lipolytic effect^13,36–38^. This may be beneficial for metabolic health in the long term, since partial inhibition of adipose tissue lipolysis may reduce systemic lipid spillover thereby attenuating ectopic lipid accumulation^39^. Further, circulating propionate was negatively associated with fasting TAG, which might be explained by the activating effect of propionate on lipoprotein lipase (LPL) in adipose tissue leading to increased TAG extraction as shown *in vitro*^40^. Furthermore, circulating butyrate was negatively associated with fasting FFA concentrations. *In vitro* data about the lipolytic effect of butyrate are contradictive showing pro- and antilipolytic effects of butyrate in white adipose tissue models^38,41^. Thus, circulating SCFA may be negatively related to systemic glycerol or FFA and/or TAG suggesting that increased circulating SCFA may reduce systemic lipid overflow with a potential beneficial effect on ectopic lipid accumulation and insulin sensitivity.

Nevertheless, with respect to markers of insulin sensitivity, neither fasting circulating nor faecal SCFA were related to fasting insulin or HOMA-IR in the total study population. Yet, fasting circulating butyrate, but not acetate and propionate, was negatively associated with fasting glucose. This is consistent with rodent studies showing that butyrate administration may have glucose lowering effects and may improve insulin sensitivity in the postprandial state^42,43^. In obesity, insulin resistance and T2DM, the abundance of butyrate-producing bacteria is reduced, which may explain to some extent the inverse association between circulating butyrate and fasting glucose in our study^44–46^.

In the subgroup analysis including prediabetic individuals with obesity, circulating acetate was negatively associated with peripheral insulin sensitivity. This is in contrast with previous rodent studies reporting a beneficial role of acetate on insulin sensitivity^34^ and with two small-scale human cross-sectional studies including obese women or morbidly obese individuals reported either none or a positive association of circulating acetate and insulin sensitivity measured via hyperinsulinemic-euglycemic clamp, respectively^47,48^. Additionally, when acetate is administered colonically, overweight participants showed increases in fasting fat oxidation, energy expenditure, and PYY secretion^12,13^, reflective of positive effects on metabolic health. Interestingly, a kinetic study showed that intravenously infused acetate remains longer in the circulation in individuals with T2DM suggesting a disturbed acetate tissue uptake and metabolism in the context of metabolic disorders^34^. Further, exogenous and endogenous acetate production but not colonic acetate absorption differed between hyperinsulinemic and normoinsulinemic individuals after rectal infusion of sodium-acetate^37,49,50^. Thus, our findings may reflect an altered endogenous acetate metabolism rather than an altered microbial-derived acetate production in metabolically compromised individuals. In contrast to fasting circulating acetate, fasting circulating propionate was positively associated with clamp-derived insulin sensitivity. Propionate has been reported to stimulate glucose uptake in 3T3-L1 adipocytes and C2C12 skeletal muscle cells *in vitro* and improve insulin sensitivity (HOMA-IR) in mice fed a high fat diet^51,52^. Possible mechanisms include an increase in peripheral glucose uptake via increased GPR41 stimulation, suppression of hepatic *de novo* lipogenesis and increase formation of beneficial odd chain fatty acids in the liver^53^.

The main limitation of our study is the cross-sectional design, which limits causal suppositions. Further, we cannot account for endogenous SCFA production, splanchnic and liver extraction or tissue utilization in this study^54,55^. Secondly, measures of GLP-1 and SCFA in the postprandial state would have been valuable. However, the study’s major strength is the availability of faecal and fasting circulating SCFA in combination with metabolic markers in a relatively large cohort with a broad range of BMI and metabolic health status. This enabled us to investigate the relationship between faecal and fasting circulating SCFA concentrations with markers of lipid and energy metabolism as well as insulin sensitivity measured by the gold standard hyperinsulinemic-euglycemic clamp. For the first time, we confirmed that fasting circulating but not faecal SCFA were related to whole-body lipolysis, fasting GLP-1 and insulin sensitivity in the fasted state. Furthermore, our study calls for urgently needed mechanistic studies in humans concerning the relationship between SCFA, GLP-1 secretion and lipid metabolism.

In conclusion, our data show that circulating but not faecal SCFA are linked to circulating GLP-1 concentrations, whole-body lipolysis and peripheral insulin sensitivity in humans. Of note, this highlights that circulating SCFA are more directly linked to metabolic health parameters. Therefore, our data indicate the need to measure circulating SCFA as a biomarker/mediator of effects on host metabolism in future human prebiotic/probiotic intervention studies. This may provide interesting leads for future research, which should aim to modulate the SCFA availability in the systemic circulation and its impact on peripheral tissue function

## Methods

### Study participants

This cross-sectional analysis included 160 Caucasian men and women aged 20–70 years with a BMI between 19.2 and 41.0 kg/m^2^ from the general population in the vicinity of Maastricht, The Netherlands during August 2013 and December 2016. Individuals had normoglycemia, impaired fasting glucose (IFG, ≥5.6 mmol/L) and/or impaired glucose tolerance (IGT, 2 hour plasma glucose of 7.8–11 mmol/L after 75 g oral glucose challenge) according to the diagnostic criteria of the American Diabetes Association, 2010^56^. Eligibility of the participants was assessed via a general health questionnaire, medical history and anthropometry during an intitial screening visit. Exclusion criteria were as follows: use of antibiotics, prebiotics, or probiotics 3 months before the study, diagnosis of T2DM, gastrointestinal or cardiovascular diseases, abdominal surgery, participants with life expectancy shorter than 5 years and participants following a hypocaloric diet. Participants did not use β-blockers, lipid- or glucose-lowering drugs, anti-oxidants, or chronic corticosteroids. All protocols were reviewed and approved by the local Medical Ethical Committee (MUMC+) and conducted in accordance with the Declaration of Helsinki (revised version, October 2008, Seoul, South Korea). Written informed consent was obtained from all participants.

### Study design

This cross-sectional analysis included metabolic parameters as well as faecal and fasting circulating SCFA concentrations of previously performed intervention studies^12,13,57–59^. In the present study, we collated and analyzed study data at baseline and thus prior to the respective interventions. In all studies, sample collection was performed after an overnight fast, and measurements were conducted according to the same standard operating procedures. Two days prior to the baseline investigation day, participants were asked to refrain from intense physical activity and alcohol consumption, and to collect a faecal sample. In the evening before the investigation day, the participants consumed a standardized low-fiber meal.

### Used data sets

The data set included baseline data from the following intervention human *in vivo* studies. These include an intervention study in prediabetic, overweight-obese individuals on the effect of antibiotics on insulin sensitivity (Clinical trial No. NCT02241421) (3), an intervention study in prediabetic, overweight-obese individuals on the effect of dietary fiber (galacto-oligosaccharides) on insulin sensitivity (Clinical trial No. NCT02271776) (4), an intervention study in normoglycemic, normal to overweight individuals on the effect of dietary fibers on gastrointestinal transit (Clinical trial No. NCT02491125) (5), and lastly two acute studies investigating the effect of different mixtures of SCFA in normoglycemic, overweight to obese individuals on human substrate and energy metabolism (Clinical trial No. NCT01826162 (6), Clinical trial No. NCT01983046 (7)).

### Baseline investigation day

After an overnight fast (>10 h), participants came to the laboratory by car or public transport. Anthropometry was measured including height, weight and waist to hip ratio. After inserting a cannula into the antecubital vein, blood samples were taken to measure plasma metabolites, hormones and inflammatory markers in the fasted state. After the blood sampling, participants were in a resting, half-supine position and fasting substrate oxidation was measured for 30 min using an open circuit ventilated hood system (Omnical, MUMC+, Maastricht, the Netherlands). Fat and carbohydrate oxidation were calculated according to the equations of Weir and Frayn^60,61^, assuming that protein oxidation accounted for 15% of total energy expenditure.

### Hyperinsulinaemic-euglycaemic clamp

Peripheral insulin sensitivity was determined in a subgroup of overweight/obese, prediabetic individuals via hyperinsulinaemic-euglycemic clamps as previously described^57,58^. In short, a cannula was inserted into an antecubital vein for infusion of glucose and insulin. To measure blood glucose, a second cannula was inserted into a superficial dorsal hand vein, which was arterialized by placing the hand into a hotbox (~50 °C). A priming dose of insulin infusion (Actrapid, Novo Nordisk, Gentofte, Denmark) was administered during the first ten min (t0–t10 min) and insulin infusion was thereafter continued at 40 mU/m^2^/min for 2 h (t10–t120 min). By variable infusion of a 20% glucose solution, plasma concentrations were maintained at 5.0 mmol/L. Peripheral insulin sensitivity (M-value, mg*(kg*min)^−1^ was calculated from the mean glucose infusion rate during the steady-state of the clamp (last 30 min, stable blood glucose concentration at 5.0 mmol/L)^62^. A high M-value represents high insulin sensitivity (i.e., more glucose needs to be infused to maintain euglycemia during insulin infusion).

### Analysis of faecal and circulating SCFA

Faecal samples were collected at home and stored in the subjects’ freezer at −20 °C maximum of two days before the baseline investigation day, transported on dry ice, and stored on arrival at the university at −80 °C. Faecal acetate, propionate, and butyrate were measured by gas chromatography-mass spectrometry (Dr. Stein and Colleague Medical Laboratory, Mönchengladbach, Germany) as previously described^63^. Plasma sample preparation for circulating SCFA analysis were performed as reported previously^64^. In short, deproteinization was performed by mixing 1 part plasma (v/v) with 2 parts methanol acidified with 1.5 mmol/l hydrochloric acid. Subsequently, samples were vortex-mixed vigorously and immediately centrifuged at 50000 × g in a model Biofuge Stratos (Hereaus, Dijkstra Vereenigde, Lelystad, the Netherlands) for 15 min. at 4 °C. 100 μl aliquots of the clear plasma supernatant were transferred into glass micro-insert vials and stored in the Combi-Pal until analysis. Samples were calibrated against external standards. The reversed phase separation was performed on a X-select ODS 2.5 µm column (150 mm × 2.1 mm I.D., Waters, Breda, the Netherlands), mounted in a Mistral Spark column oven (Separations, H.I. Ambacht, the Netherlands), set to 45 °C. Samples were completely separated from other components into the individual SCFA in a 25 min. gradient cycle between an aqueous 1 mmol/l solution of sulfuric acid and ethanol. Post-column, the solvent pH was enhanced to about 9, by mixing with 150 mmol/l ammonia in ethanol to maximize negative ionization. Samples were processed using a Combi-PAL sample processor (Interscience, Breda, the Netherlands) with Peltier chilled sample storage compartments set to 10 °C. The system was equipped with a 50 µl sample loop. Separated SCFA were detected using a model LTQ XL linear ion trap mass spectrometer (Thermo Fisher Scientific, Breda, the Netherlands), equipped with an ion-max electrospray probe. The MS was operated in MS-MS full scan negative mode.”

### Blood collection and biochemical analysis

Blood was collected in pre-chilled EDTA tubes (0.2 mol/L EDTA; Sigma, Dorset, UK) for SCFA, insulin, glucose, FFA, TAG, free glycerol, LBP, GLP-1, TNF-α, IL-6 and IL-8 analyses during fasting conditions. For GLP-1 and PYY analysis, 20 μl of dipeptidyl peptidase-IV inhibitor (Milipore Merck, Billerica, MA, USA) was added to EDTA and Aprotinin (Becton Dickinson, Eysins, Switzerland) tubes, respectively. Samples were centrifuged at 3500 g, 4 °C for 10 minutes; plasma was aliquoted and directly snap-frozen in liquid nitrogen and stored at −80 °C until analysis. Plasma glucose concentrations were determined using commercially available reagent kit (Glucose Hexokinase CP, Horiba ABX Pentra, Montpellier, France) involving a two-step enzymatic reaction with hexokinase followed by Glucose-6-phosphate-dehydrogenase resulting in D-gluconate-6-phosphate. The colorimetric reaction was measured using an automated spectrophotometer (ABX Pentra 400 autoanalyzer, Horiba ABX Pentra). Plasma FFA concentrations were measured using a commercially available kit (NEFA-HR(2) assay, Wako, Sopachem BV, Ochten, the Netherlands) with a two-step enzymatic reaction involving acylation of Coenzyme(Co) A followed by acyl-CoA oxidase resulting in the production of hydrogen peroxide as substrate that in the presence of peroxidase yields a blue purple pigment, measured with a colorimetric reaction using an automated spectrophotometer (ABX Pentra 400 autonalyzer, Horiba ABX Pentra). Plasma TAG were determined using a commercially available kit (Triglycerides CP, Horiba ABX Pentra) based on enzymatic reactions involving lipoprotein lipase, glycerolkinase and glycerol-3-phosphate oxidase resulting in the production of hydrogen peroxide as substrate of a colorimetric reaction measured using the automated spectrophotometer (ABX Pentra 400 autonalyzer, Horiba ABX Pentra). Plasma glycerol was measured after precipitation with an enzymatic assay (Enzytec TM Glycerol, Roche Biopharm, Basel, Switzerland) involving phosphorylation of glycerol to L-glycerol-3-phosphate by glycerokinase and the colorimetric reaction is measured using an automated spectrophotometer (Cobas Fara, Roche Diagnostics, Basel, Switzerland). Plasma insulin was determined with a commercially available radioimmunoassay (RIA) kit (HI-14K Human Insulin specific RIA, Millipore Merck) according to the manufacture’s protocol. Plasma IL 6, IL-8 and TNF-α were determined with an commercialy available enzyme-linked immunosorbent assay (ELISA) kit (Human Proinflammatory II 4-Plex Ultra-Sensitive kit, Meso Scale Diagnostics, MD, USA). Plasma samples were assayed for total GLP-1 immunoreactivity using an antiserum that reacts equally with intact GLP-1 and the primary (N-terminally truncated) metabolite as previously described^65^. PYY concentrations were determined using a commercially available RIA kit (Human PYY (3–36) Specific RIA, Millipore Merck). Plasma LBP was measured as previously described^66^. In short, plates (Greiner Mocrolon 600 high binding; Sigma Aldrich, St. Louis, MO) were coated with polyclonal anti-human LBP antibodies. Diluted plasma samples (1:5000) and a standard dilution series with recombinant LBP were added to the plate. Detection occurred with a biotinylated polyclonal rabbit anti-human LBP IgG, followed by peroxidase-conjugated streptavidin and substrate. The detection limit for the LBP assay was 200 pg/ml.

### Statistical analysis

Normality of data was assessed with the Gaussian distribution and Kolmogorov-Smirnov procedure, and ln or Z-score transformation was used if assumption of normality was not met. HOMA-IR was calculated as previously described^67^. In case of missing data, the participant was excluded from the analysis. Multicollinearity was checked using variance inflation factor index <10. First, we used simple linear regression to investigate the associations between faecal and circulating concentrations of acetate, propionate and butyrate (as dependent variables) and metabolic parameters (as independent variables) i.e. insulin sensitivity (M-value), insulin resistance (HOMA-IR), circulating glucose, insulin, circulating lipids (TAG, FFA and glycerol), circulating inflammatory markers (IL-6, IL-8, TNF-α and LBP) and fasting substrate oxidation. Subsequently, we used multiple linear regression to test whether the associations between faecal and circulating SCFA and the aforementioned metabolic parameters were independent of the covariates sex, age and BMI. All data were analysed using SPSS 22.0 (IBM, Armok, U.S.) with significance set at P < 0.05.

### Ethics approval

The studies summarized in this manuscript were approved by the Medical Ethics Committee of Maastricht University Medical Centre and was conducted according to the ethical standards of the Helsinki Declaration and in accordance with the Medical Research Involving Human Subjects Act (WMO). All patients provided verbal and written informed consent.

## Supplementary information

Supplementary Figure

## Data Availability

The used intervention study data are unsuitable for public deposition due to ethical restrictions and privacy of participant data. Data are available from these studies for any interested researcher who meets the criteria for access to confidential data. Prof. Ellen Blaak (e.blaak@ maastrichtuniversity.nl) may be contacted to request study data.
