# Supplementary material for: Circulating but not faecal short-chain fatty acids are related to insulin sensitivity, lipolysis and GLP-1 concentrations in humans
Source: Sci Rep. 2019 Aug 29;9:12515. doi: 10.1038/s41598-019-48775-0 (PMC6715624; doi:10.1038/s41598-019-48775-0)
Supplement: Supplementary file 1 — Supplementary Figure [file 41598_2019_48775_MOESM1_ESM.pdf]

Supplementary for “*Circulating but not faecal short-chain fatty acids are related to insulin sensitivity, lipolysis and GLP-1 concentrations in humans*”

SUPPLEMENTARY FIGURE 1

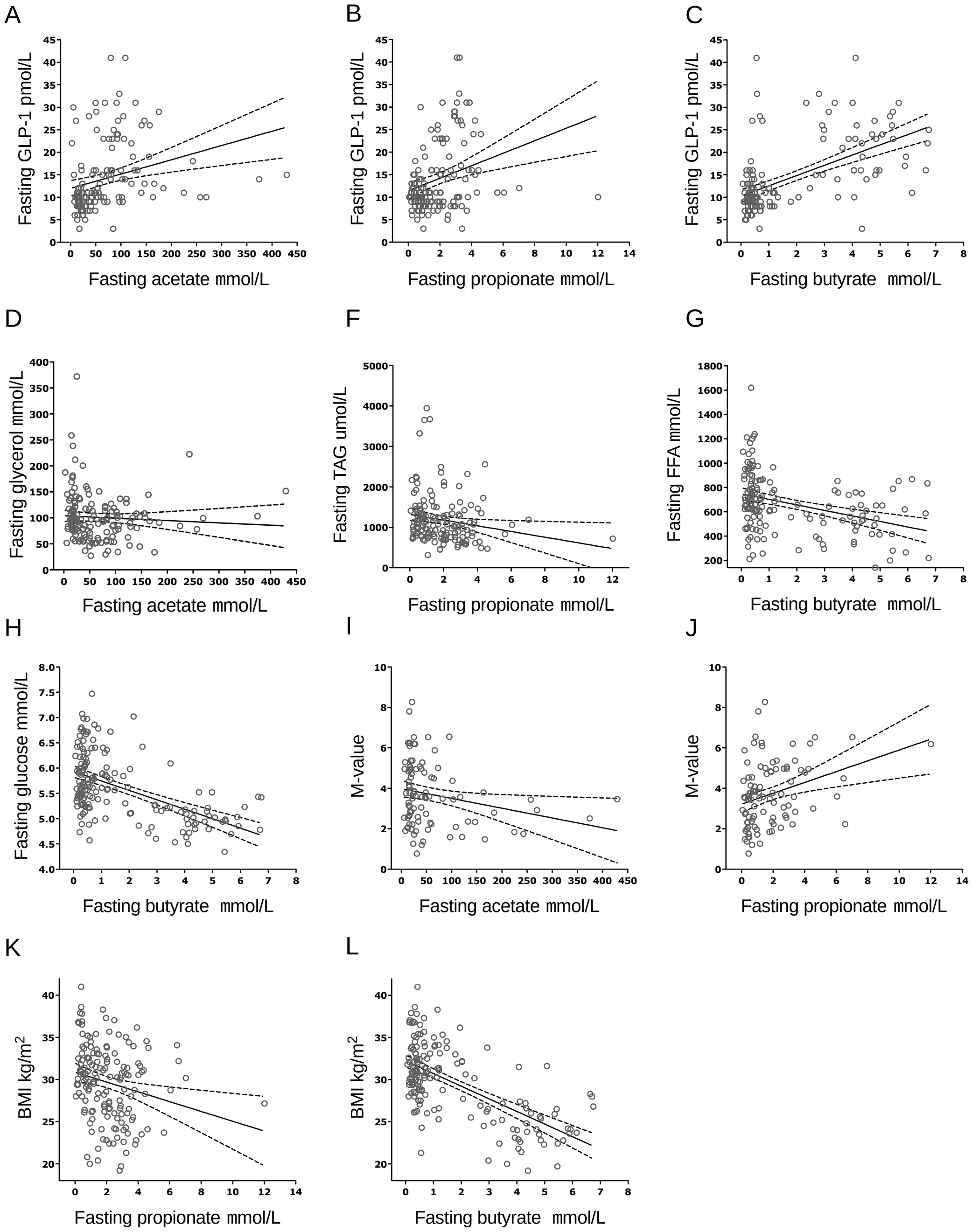


**Supplementary Figure 1** Associations between metabolic parameters, BMI and insulin sensitivity and fasting circulating SCFA. Linear regression scatter plots of fasting circulating SCFA with fasting GLP-1 (**A-C**), fasting acetate with fasting glycerol (**D**), fasting propionate with fasting TAG (**F**), fasting butyrate with FFA (**G**) and fasting glucose (**H**), fasting acetate with M-value (**I**), fasting propionate with M-value (**J**) and BMI (**K**) and fasting butyrate with BMI (**L**). GLP-1 glucagon like peptide 1, TAG triacylglycerol, FFA free fatty acids, BMI body mass index
